# Different Astrocytic Activation between Adult *Gekko japonicus* and Rats during Wound Healing *In Vitro*

**DOI:** 10.1371/journal.pone.0127663

**Published:** 2015-05-28

**Authors:** Yun Gu, Jian Yang, Haijiao Chen, Jing Li, Man Xu, Juan Hua, Jian Yao, Yongjun Wang, Yan Liu, Mei Liu

**Affiliations:** 1 Jiangsu Key Laboratory of Neuroregeneration, Co-innovation Center of Neuroregeneration, Nantong University, Nantong, Jiangsu, 226001, China; 2 Department of Histology and Embryology of Medical College, Nantong University, Nantong, Jiangsu, 226001, China; University of Toronto, CANADA

## Abstract

Glial scar formation is a major obstacle to regeneration after spinal cord injury. Moreover, it has been shown that the astrocytic response to injury differs between species. *Gekko japonicas* is a type of reptile and it shows differential glial activation compared to that of rats. The purpose of the present study was to compare the proliferation and migration of astrocytes in the spinal cords of geckos and rats after injury *in vitro*. Spinal cord homogenate stimulation and scratch wound models were used to induce astrocytic activation in adult and embryonic rats, as well as in adult geckos. Our results indicated that astrocytes from the adult rat were likely activated by mechanical stimulation, even though they showed lower proliferation abilities than the astrocytes from the gecko under normal conditions. Furthermore, a transcriptome analysis revealed that the differentially expressed genes in astrocytes from adult rats and those from geckos were enriched in pathways involved in proliferation and the response to stimuli. This implies that intrinsic discrepancies in gene expression patterns might contribute to the differential activation of astrocytes between species.

## Introduction

After spinal cord injury, regeneration and functional recovery of the damaged area always fails in adult mammals. Major reasons for the constant failure include the weak ability of axons to regenerate, as well as the glial scar [1–3]. Many recent studies have suggested that the injured axons of embryonic or adult members of warm-blooded species are still capable of regeneration when glial scarring is inhibited [4]. This indicates that the astroglial response is crucial for successful regeneration of the spinal cord.

Following spinal cord injury, activated astroglia up-regulate the expression of intermediate filament proteins (such as glial fibrillary acidic protein [GFAP]), proteoglycans, and other molecules that are inhibitory to axon growth. Furthermore, the reactive astrocytes deposit extracellular matrix molecules (e.g., chondroitin sulfate proteoglycan[CSPG]) and form the glial scar [5]. Early astroglial activation plays an important role in the repair process because it helps re-establish homeostasis by insulating the injury site and restoring the integrity of the blood-brain barrier [6]. However, when a glial scar is formed, it also presents a physical and biochemical barrier that inhibits damaged axons from regenerating and reestablishing functional connections.

Astroglial activation is differentially responsive depending on the species. For example, in zebrafish, astrocytes can migrate and bridge the axon, thus extending through the lesion site after spinal cord transection [7]. In amphibians, regenerating axons can also pass through the glial scar after injury [8]. This phenomenon was found not only in lower vertebrates, but also in fetal or embryonic mammals [9]. These studies imply that astrocytes might promote an environment that permits axon extension in lower vertebrates and embryonic mammals, while in adults, astrocytes produce an environment that increases scar formation and inhibits neurite growth.

In the current study, we wanted to understand the mechanisms that underlie the different astrocytic responses to injury. Astroglial activation has been investigated in fish, amphibians, and mammals; however, few studies have been conducted in reptiles. *Gekko japonicus* is a member of the reptile family, and is a feasible animal model for studying regeneration because of its remarkable capacity for tail restoration [10]. Here, we observed that the astrocytic responses to spinal cord injury in geckos were different from those in rats. To elucidate the mechanisms underlying these variable astroglial responses, we isolated astrocytes from adult geckos (A-Gecko AS), adult rats (A-Rat AS), and embryonic rats (E18-Rat AS), and analyzed the factors released, as well as the extracellular matrix of the different astrocytes in an *in vitro* wound healing model. We further investigated transcriptomes of the three groups of astrocytes (A-Gecko AS, A-Rat AS, E18-Rat AS) in hopes that we might discover the molecules and signaling pathways that contribute to the variability of astroglial activation between different species.

## Materials and Methods

### Animal and injury model

Adult Sprague–Dawley rats, embryonic Sprague–Dawley rats (E18), and adult *G*. *japonicas* were obtained from the Experimental Animal Center of Nantong University. The *G*. *japonicas* used in this study were mature individuals and all were 3±0.5g in weight with an average total body length (snout to vent) of 5.2 cm, including males and females. They were freely fed with mealworms and water and housed in an air-conditioned room with a controlled temperature (22–25°C) and saturated humidity. All experimental protocols applied to the animals were approved by the Laboratory Animal Care and Use Committee of our medical school. Animal models of spinal cord injury were established using procedures described previously [11]. Briefly, rats were anesthetized with 1% pentobarbital sodium, and a laminectomy was applied at the T9 to T10 spinal segment level. The dura was removed and the T10 spinal segment was completely transected using ophthalmic scissors. Geckos received a complete spinal cord transection at the L10–11 lumbar vertebrae level using the same procedure. The severed ends of the cord typically retracted 3–5 mm (rats) or 1–2 mm (geckos) and were inspected under a surgical microscope to ensure complete transection. Incisions were then closed, and animals were placed in a temperature- and humidity-controlled chamber overnight. Animals were returned to their cages and allowed to recover for 1 or 4 weeks before being sacrificed. For sham-operated controls, animals underwent a laminectomy without transection.

### Immunohistochemistry and Nissl staining

Control and injured animals were sacrificed at scheduled times post-injury, and were perfused intracardially with 4% paraformaldehyde (PFA) in 0.1 mol/L phosphate buffer solution (PBS). Spinal cords were removed and post-fixed overnight at 4°C. Fixative was then replaced with 20% sucrose for 2–3 days and then 30% sucrose for 2–3 days. Following cryoprotection, spinal cords were embedded in optimum cutting temperature compound (Sakura Finetek Tissue-Tek, USA), and 10-μm frozen cross-sections of the spinal cord (2 mm above or below the injury epicenter) were prepared. Sections were blocked in 10% goat serum with 0.3% TritonX-100 and 1% (w/v) bovine serum albumin for 2 h at room temperature, followed by overnight incubation at 4°C with polyclonal anti-GFAP antibody (1:200; Dakocytomation, CA, USA). The next day, sections were reacted with fluoresce in isothiocyanate (FITC)-conjugated secondary antibodies for 2 h at room temperature, and then examined with a DMR fluorescent microscope (Leica Microsystems, Wetzlar, Germany). For Nissl staining, prepared frozen sections were mounted onto poly-l-lysine-coated slides, dehydrated with ethanol, and then treated with xylene for 5 min. After being washed with double-distilled water, sections were incubated with 1% cresyl violet (Sigma-Aldrich, St. Louis, MO, USA) solution for 5 min at 50°C and then dehydrated with ethanol.

### Astrocyte culture

Primary astrocytes were prepared from the spinal cords of both adult and E18 rats, as well as from adult geckos. Spinal cords without the meninges were enzymatically dissociated and the dispersed cells were seeded on a 6-cm dish with a density of 1 × 10^5^ cells/mL in Dulbecco's Modified Eagle's Medium (DMEM)/F-12 medium with 10% fetal bovine serum (FBS) and 1% P-S (Penicillin 100 units/mL, Streptomycin 100 μg/mL). The osmotic pressure of the culture media for rats was 300–350 mmol/kg [1, 2]. All of the same components were used for the rat and gecko culture procedures, except that the osmotic pressure of the culture media for the geckos was 200–260 mmol/kg. Cultures were incubated with 5% CO_2_ at 37°C for rats or at 30°C for geckos. Astrocyte cultures were examined for cell cycle by flow-cytometric analysis after synchronization by serum deprivation for 48 h [12]. Synchronized astrocytes of passage 3 were used in this study. The purity of the astrocytes isolated from geckos and rats was determined with GFAP immunostaining, and their proliferation rates were evaluated with bromodeoxyuridine (BrdU) incorporation assays.

### Models of astrocytic activation

#### Spinal cord homogenate (SCH) stimulating model

Spinal cords from geckos and rats without the meninges were placed into sterile dishes, rinsed with ice-cold PBS, and homogenized in serum-free DMEM (100 μg/mL) on ice. The tissue was then centrifuged at 12000 rpm for 15 min at 4°C, and the supernatant was obtained as spinal cord homogenate (SCH). SCH was then quantified by the BCA protein assay kit (Pierce, Rockford, IL, USA), and the final concentrations of 5.0 and 50 μg/mL were used to stimulate cultured astrocytes in a 96-well dish. After the 24-h treatment, cell proliferation was detected by BrdU-enzyme-linked immunosorbent assays (ELISAs).

#### Scratch wound model

A scratch wound was made by scraping the cell monolayer with a sterile 100-μL pipette, as described previously [13, 14]. We changed the culture medium immediately after scraping to prevent the medium from being conditioned with cell debris and factors released from the detached cells. Wounded cultures were then incubated in DMEM/F-12 and 10% FBS for 4, 24, or 48 h until the following studies were conducted. At the indicated times, migrating cells at the wound area were photographed using a DMR inverted microscope (Lexica Microsystems, Germany), and the percentage of wound healing was measured using Image-Pro Plus version 6.2 software (Media Cybernetics, Rockville, MD, USA). Assays were performed three times using triplicate wells.

### BrdU-ELISA assay

BrdU labeling solution (10μL/well) was added into 100μLof culture media (final concentration was 10 μM BrdU). Cells were then re-incubated for 24 h and run through the Cell Proliferation ELISA protocol using the BrdU Kit (Roche, USA). Optical density (OD) values were obtained by measuring the absorbance at 450 nm with a micro-plate reader (BioTek, Winooski, VT, USA). Measurements were repeated at least three times.

### Immunofluorescence

After scraping the culture, astrocytes were fixed by 4% PFA at different time points, and incubated with primary antibodies (anti-GFAP antibody, 1:500; Dakocytomation; or anti-chondroitin sulfate antibody [CS-56], 1:200, Abcam) at 4°C overnight. Astrocytes were then rinsed with PBS, and incubated with a secondary antibody (FITC-goat anti-rabbit polyclonal, TRITC-goat anti-mouse polyclonal, Sigma) for 2 h at room temperature. Nuclei were counterstained with 4', 6-diamidino-2-phenylindole. To measure the proliferation of astrocytes, BrdU (1:1000) was added to the culture medium at the different time points after the scraping injury (12, 24, and 48 h). Procedures were performed according to instructions described in the 5-Bromo-2′-deoxy-uridine Labeling and Detection Kit I (Roche, USA). Representative sections were then observed with epifluorescence using a Leica microscope.

### Cell cycle and cell migration assays

The cell cycle assay was performed as described in our previous study [15]. Briefly, cells (5 × 10^5^) were trypsinized, fixed in cold 70% ethanol for at least 1 h, and stored at -20°C. The fixed cells were then washed with PBS, treated with RNase (1 mg/mL), and stained with propidium iodide (50 mg/mL) for 30 min at 4°C, after which DNA content analysis was performed on an EPICS ELITE flow cytometer (Beckman Coulter, USA). The cell cycle distribution was analyzed using ModFit LT2.0 software. Astroctye migration was examined using 6.5 mm transwell chambers with 8-μm pores (Costar, Cambridge, MA, USA). A 100-μL medium containing dissociated astrocytes of 2 × 10^4^ was then transferred to the top chambers of each transwell, and 600 μL of complete medium was added into the lower cell-free chambers. After allowing the cells to migrate for 16 h, non-migrated cells on the upper surface of each membrane were cleaned with a cotton swab. Cells adhering to the bottom surface of each membrane were stained with 0.1% crystal violet, imaged, and counted using a DMR inverted microscope (Leica Microsystems, Germany). Assays were performed three times using triplicate wells.

### RNA sequencing assays of cultured astrocytes from adult rats, geckos, and E18 rat spinal cords

#### RNA sequencing for differentially expressed genes

Total RNA was extracted by the Trizol reagent (Invitrogen, USA) from astrocytes of adult rats, geckos, and E18 rat spinal cords, which were cultured using the methods described above. Beads with oligo (dT) were used to isolate poly(A) mRNA after the total RNA was collected. Fragmentation buffer was then added to cut the mRNA into short fragments, which were used as templates. Random hexamer primers were used to synthesize first-strand cDNA, while second-strand cDNA was synthesized using a mixture of buffer, dNTPs, RNase H, and DNA polymerase I. Short fragments were purified with QiaQuick polymerase chain reaction (PCR) extraction kits and resolved with elution buffer for end repair and the addition of poly(A). Next, the short fragments were connected with sequencing adaptors. For amplification with PCR, we selected suitable fragments as templates based on agarose gel electrophoresis. Finally, libraries were sequenced using IlluminaHiSeq2000 (Illumina, USA).

Using the SOAP program (BGI-Shenzhen, China) [16], clean reads were mapped to the reference genomes and gene sequences. No more than five mismatches were allowed in the alignment. Proportions of the clean reads were then mapped back to the genome and genes, providing an overall assessment of the sequencing quality.

Annotation of the transcriptome was obtained by blasting the reference sequences that we already had. We defined gene coverage as the percentage of a gene covered by the reads, which was equivalent to the ratio of the number of bases in a gene covered by unique mapping reads to the number of total bases in that gene. The calculation of gene expression used the reads per kilobase of transcript per million reads mapped (RPKM) method [17], as follows:

$$RPKM=\frac{10^{6}C}{NL/10^{3}}$$

There were 5230 differentially expressed genes (DEGs) between the cells from the embryonic rat and the cells from the adult rat, including 54 DEGs without any annotation. These DEGs were used as a benchmark for examining possible expression patterns in the three sample groups.

#### Gene ontology (GO) and functional enrichment analyses

GO and functional enrichment analyses mapped all genes to GO terms in the database (http://www.geneontology.org/) by calculating gene numbers for every term [18]. We then used an ultra-geometric test to find significantly enriched GO terms in the target gene list compared to the genomic background using the following formula:

$$P=1-\sum_{i=0}^{m-1}\frac{\left(\begin{array}{l} M\\ i \end{array}\right)\left(\begin{array}{l} N-M\\ n-i \end{array}\right)}{\left(\begin{array}{l} N\\ n \end{array}\right)}$$

where *N* denotes the total number of genes with a GO annotation, and *n* is the number of target genes in *N*. *M* indicates the total number of genes that are annotated to certain GO terms, and *m* is the number of target genes in *M*. The calculated *p*-value went through Bonferroni correction with a threshold-corrected *p*-value of ≤0.05. GO terms fulfilling this condition were considered to have significantly enriched GO terms.

#### Kyoto encyclopedia of genes and genome-based (KEGG) pathway enrichment analyses

The Kyoto encyclopedia of genes and genome-based (KEGG) pathway is the major public pathway-related database. We used the pathway enrichment analysis to identify significantly enriched metabolic pathways or signal transduction pathways in the target gene list compared with the whole genomic background [19]. We employed the same formula that was used for the GO analysis, but *N* was the number of all genes in the KEGG annotation, *n* was the number of genes in *N*, *M* was the number of all genes annotated to specific pathways, and *m* was the number of genes in *M*.

#### Real-time quantitative PCR analysis

The total RNA was isolated from cells using the methods described above. The first-strand cDNA was synthesized using an Omniscript Reverse Transcription Kit (Qiagen, Netherlands) in a 20-μL reaction system containing 2 μg total RNA. The 1-μL aliquot of the first-strand cDNA was amplified using primers designed to investigate the expression of target genes by real-time PCR. The reaction mixtures included 10 μL of 2× Fast Evagreen qPCR Master Mix (Biotium, USA), 2 μL of 10× ROX (Biotium), gene-specific primers at a final concentration of 0.5 μM, and 1 μL of cDNA. Real-time PCR was performed in a StepOne real-time PCR system (ABI Applied Biosystems, USA). The thermal cycling program consisted of 2 min at 96°C, followed by 45 cycles of 15 s at 96°C and 1 min at 60°C. Data collection was performed during the 60°C extension step. To account for variability in the total RNA input, the expression of the target genes was normalized to that of the *EF1α* gene. In addition, a negative control without first-strand cDNA was prepared. The relative expression was calculated using the comparative 2^−ΔΔCt^ method.

### Statistical analysis

Data are expressed as the mean ± the standard deviation (SD). Differences between groups were analyzed by one-way analyses of variance (ANOVAs) using the SPSS software (IBM, USA). Statistical significance was set at *p*< 0.05. In this study, each experiment was repeated at least three times.

## Results

### Differences between adult *G*. *japonicus* and adult rats in terms of astroglial activation following spinal cord injury

**Fig 1:**
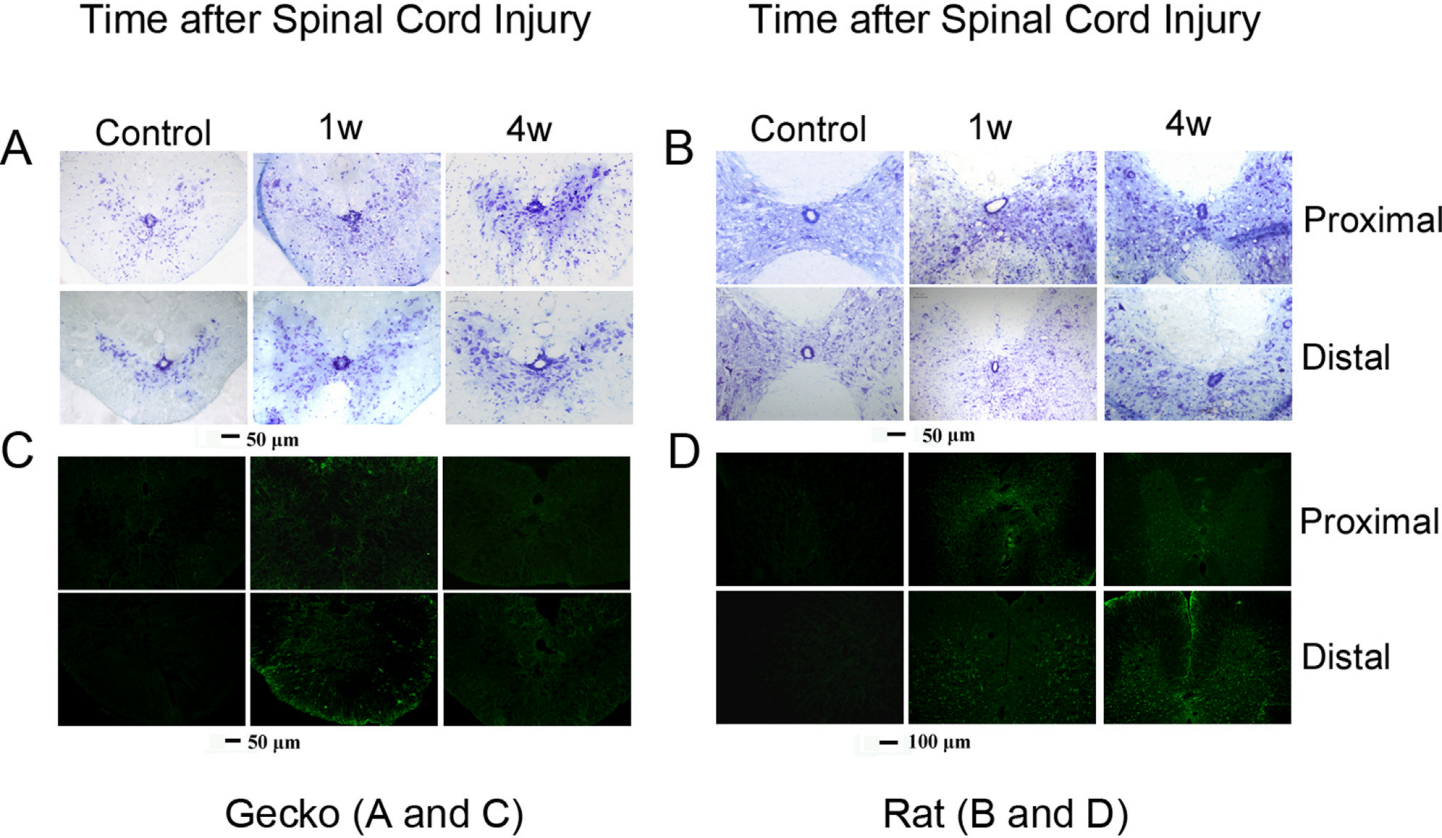
Gliosis after spinal cord injury is different in rats and *Gekko japonicus*. Nissl staining (A, B) and glial fibrillary acidic protein (GFAP) immunehistochemical analysis (C, D) of adult geckos (A, C) and adult rats (B, D) after spinal cord transection (n = 6 per group). GFAP staining is shown in panels C and D. GFAP expression in activated astroglia was observed at 1 week post-injury in both adult geckos and rats. In geckos, the GFAP level was weakened at 4 weeks post-injury, while GFAP in adult rats was still strong 4 weeks post-injury.

In all animals, the spinal cord was transected at the superior border of the intumescentia lumbalis. In *G*. *japonicus* this region was on the 10^th^ lumbar vertebra, while in the rat this area was on the 9^th^ thoracic vertebra. Cross sections from tissue located one vertebra away from the injury site were prepared for Nissl and GFAP staining. Nissl staining was employed to observe gray and white matter morphology in the proximal and distal stumps. GFAP, an intermediate filament, was used because of its involvement in the formation of glial scars [20]. In adult rats, GFAP expression continuously increased until 4 weeks after spinal cord injury, while in *G*. *japonicus* GFAP expression was higher at 1 week after SCI and decreased 4 weeks after the operation (Fig 1). The results are consistent with the previous reports about the GFAP expression pattern after spinal cord transection [21, 22], and indicate that astroglial activation was attenuated in adult *G*. *japonicus* compared to in adult rats.

### The different astrocytic responses to external stimuli between species may have endogenous origins

Spinal cord injury induced astrocytic activation in all of the animal models used, however, differences were observed between astrocytes derived from *G*. *japonicus* and those derived from adult rats (Fig 1). We wanted to explore the cause of this difference; more specifically, whether it was caused by the astrocytes or by an external stimulus. It is well known that astrocytes are activated following spinal cord injury [23]. Therefore, we prepared SCHs from both adult geckos and adult rats, which served as the external stimuli, and further tested whether these homogenates could stimulate the cell proliferation of astrocytes in *G*. *japonicus* or in rats. The SCHs (with concentrations of 5 and 50 μg/mL) of adult geckos were applied to astrocytes from adult geckos or adult rats, while the SCHs (with concentrations of 5 and 50 μg/mL) of adult rats were applied to astrocytes from adult geckos or adult rats. A BrdU-ELISA assay was then used to determine the cell proliferation of gecko and rat astrocytes after SCH treatment. The OD values were also obtained.

**Fig 2:**
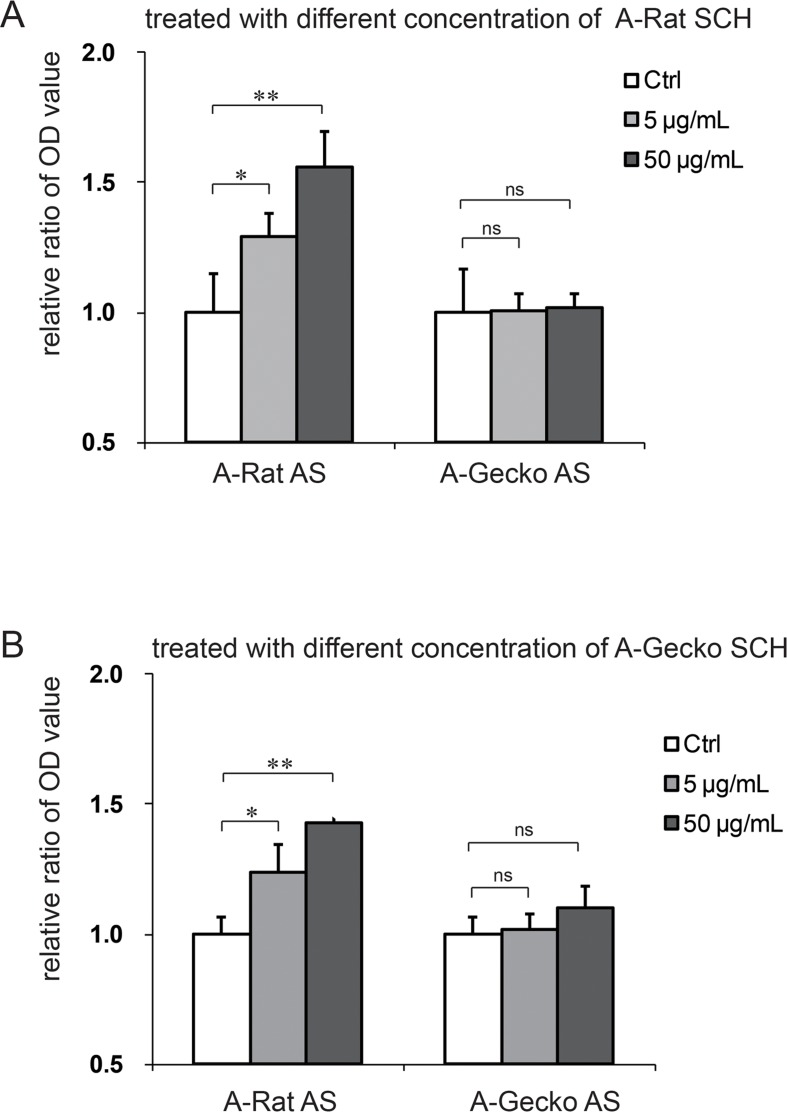
Spinal cord homogenates (SCHs) stimulated the proliferation of rat astrocytes, but not gecko astrocytes. Different concentrations of SCHs from adult rats stimulated the proliferation of cultured adult rat astrocytes (A-Rat AS), but had no effect on the proliferation of cultured adult gecko astrocytes (A-Gecko AS) (A). SCHs from *Gekko japonicus* stimulated the proliferation of A-Rat AS, but had no effect on A-Gecko AS (B). The optical density (OD) value was measured, and data are represented as the mean ± the standard deviation (SD). Data were normalized, as described in the results. **p < 0.01 vs. control; *p < 0.05 vs. control; ns = no significant change vs. the control.

Given that the proliferation rates of rat and gecko astrocytes might be different, the capacity of these astrocytes to respond to the extrinsic stimulus should be evaluated with the ratio (proliferation rate after treatment/proliferation rate before treatment) variation. The OD values of the adult rat astrocytes (A-Rat AS) were normalized to 1, and the OD values in the A-Rat AS treated with SCH were calculated by dividing by the OD value of control A-Rat AS. The data for the cell proliferation in gecko astrocytes upon SCH treatment were handled in the same way. As shown in Fig 2, we found that the proliferation of A-Rat AS increased in a dose dependant manner when treated with rat SCHs (control = 1 ± 0.15, SCH [5 μg/mL] = 1.29 ± 0.09, SCH [50 μg/mL] = 1.56 ± 0.13). However, no difference was observed when adult gecko astrocytes (A-Gecko AS) were treated with rat SCHs (control = 1 ± 0.17, SCH [5 μg/mL] = 1.01 ± 0.07, SCH [50 μg/mL] = 1.02 ± 0.06). A-Rat AS also showed differences when they were treated with gecko SCHs (control = 1 ± 0.07, SCH [5 μg/mL] = 1.24 ± 0.11, SCH [50 μg/mL] = 1.40 ± 0.08). However, treating A-Gecko AS with gecko SCHs yielded no difference (control = 1 ± 0.07, SCH [5 μg/mL] = 1.02 ± 0.06, SCH [50 μg/mL] = 1.10 ± 0.08). In sum, these results show that the proliferation of rat astrocytes was promoted by different concentrations of SCHs (5 and 50 μg/mL) from either adult rats or geckos, while no notable changes were observed when gecko astrocytes were treated with the SCHs of adult rats or geckos (Fig 2). Thus, we speculate that the different astrocytic responses to the external SCH stimuli might be due to the endogenous properties of the astrocytes themselves.

### *In vitro* activation of gecko astrocytes differs from the astrocytic activation of adult rats

Next, we investigated whether astrocytic activation in geckos and rats differed when using the *in vitro* wound healing model. The above data indicate that the intrinsic properties of astrocytes from geckos are different from those of rats, suggesting phylogenetic discrepancies in the astrocytes from different species. It is well known that the ability to regenerate after spinal cord injury is observed not only in lower vertebrates, but also in fetal or embryonic mammals. Thus, we also wanted to know if astrocytes from embryonic rats (E18-Rat AS) are more similar to the A-Gecko AS or to the A-Rat AS.

**Fig 3:**
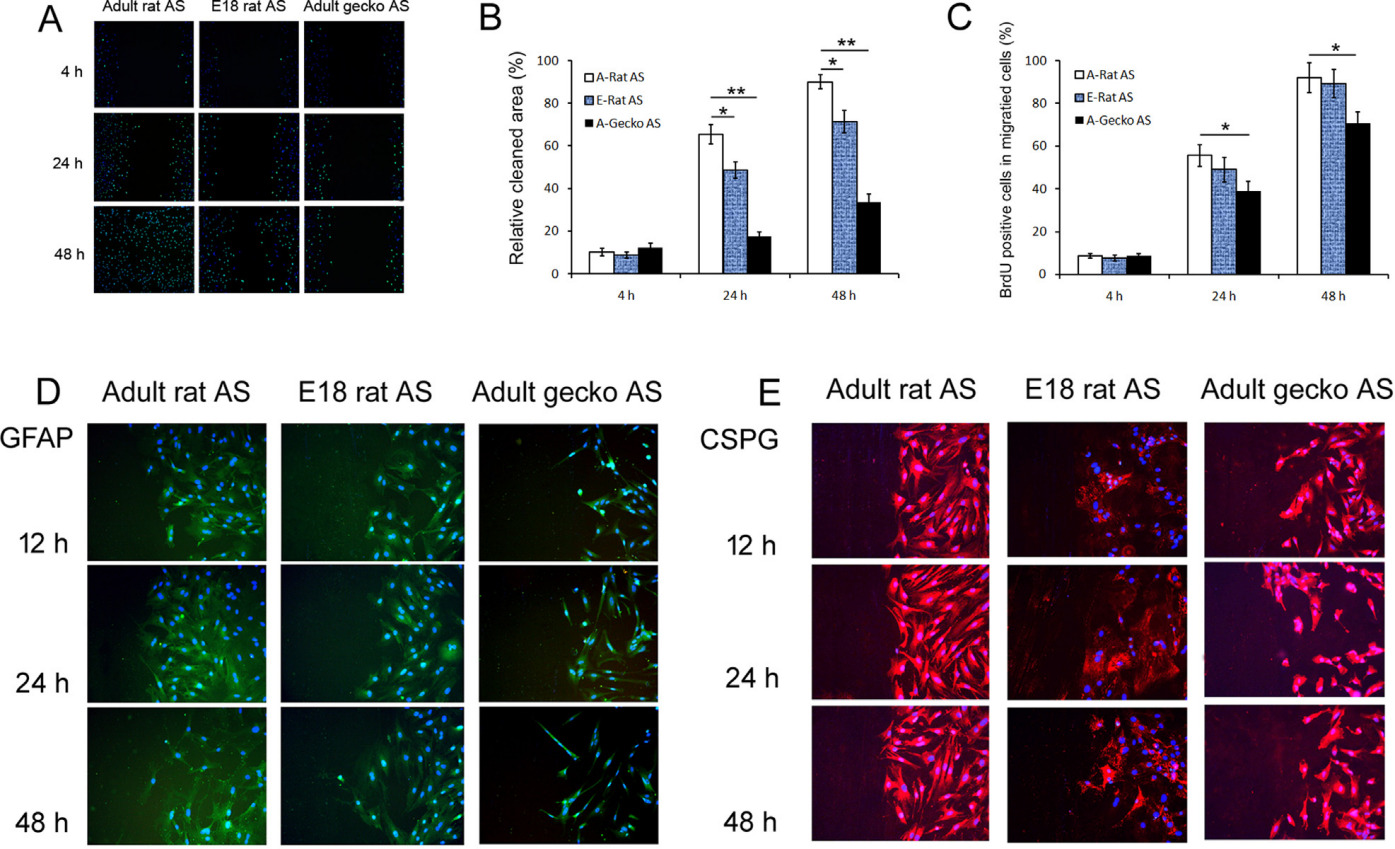
Astrocytic responses from adult and embryonic rats and geckos after *in vitro* scratch wound. The responses in adult rat astrocytes (A-Rat AS) were different from that of astrocytes from embryonic rats (E18-Rat AS) and adult geckos (A-Gecko AS). (A) Representative images of wound healing combined with BrdU assays at 4, 24, and 48 h. A-Gecko AS showed obvious delays in covering the wound and decreased proliferation abilities compared with A-Rat AS. For E18-Rat AS, the capacity for proliferation and migration was between that of adult rats and geckos. (B) Graph showing the percentage of cell migration in the cleaned space at 4, 24, and 48 h after the scratch wounding. (C) Graph showing the percentage of BrdU-positive cells that migrated into the cleaned space at 4, 24, and 48 h after the scratch wounding. Data are represented as the mean ± SD. **p < 0.01, *p < 0.05 vs. A-Rat AS. (D, E) the expression of GFAP and CSPG after scratch injury. GFAP expression in astrocytes from the adult rat, embryonic rat, and adult gecko increased following injury. The extent of the increase was highest in A-Rat AS, and lowest in A-Gecko AS. In addition, GFAP expression ceased to increase after 24 h in E18-Rat AS and A-Gecko AS, while GFAP expression remained high in A-Rat AS. CSPG expression was observed in all three groups, but did not show obvious variations after injury.

In order to address this, we performed scratch assays combined with BrdU to evaluate the capacity of proliferation and/or migration of different astrocytes that contribute to gliosis after spinal cord injury. As shown in Fig 3A and 3B, 4 h after we induced a scratch wound, A-Gecko AS exhibited no significant difference in the percent of wound healing (12.3% ± 1.9) compared to the rat astrocytes (A-Rat AS = 10.1% ± 1.7; E18-Rat AS = 8.8% ± 1.4). This pattern changed at 24 h after the scratch wound introduced. A-Gecko AS exhibited obvious delays in the percent of wound healing (A-Rat AS = 65.3% ± 4.5, E18-Rat AS rat = 48.7% ± 3.8, and A-Gecko AS = 17.6% ± 2.0). After 48 h, when the adult rat astrocytes fully covered the wounded zone (90% ± 3.3), the A-Gecko AS had just begun to occupy the wounded region (33.7% ± 3.8). The migration abilities of E18-Rat AS were between those of adult rats and geckos, and showed 71.3% (± 5.1) recovery.

Results of the BrdU assay showed (Fig 3A and 3C) that, at 4 h after the scratch wound, there was no difference in labeling between the astrocytes of all three groups (adult rat = 8.6% ± 1.2; E18 rat = 7.5% ± 1.3; adult gecko = 8.7% ± 1.1). At 24 and 48 h after injury, the percentages of BrdU-positive cells in A-Rat AS (55.6% ± 5.1 and 91.8% ± 7.0, respectively) were significantly increased compared to the A-Gecko AS (38.8% ± 4.6 and 70.6% ± 5.4, respectively). Thus, at 24 and48 h after scratch injury the proliferation of A-Gecko AS was reduced by 30.2% and 23.1%, respectively, when compared to the proliferation of A-Rat AS. However, no significant changes in astrocytic proliferation were observed between adult rats and E18 rats after the scratch wound (at 24 and 48 h, percentage of proliferating A-Rat AS = 55.6% ± 5.1 and 91.8% ± 7.0, respectively; E18-Rat AS = 49.0% ± 5.8 and 89.2% ± 7.0, respectively).

Increased expression of the intermediate filament GFAP and extracellular matrix molecules (such as CSPG) is a well-known index of astrocytic activation [2]. Therefore, we determined the expression patterns of these two molecules in astrocytes derived from all three animal models after the scratch injury. We found that GFAP expression in the three groups increased, and that the extent of the increase was highest in the A-Rat AS, and lower in the E18-Rat and A-Gecko AS (Fig 3D). In addition, GFAP expression ceased to increase after 24 h in E18-Rat and A-Gecko AS, while GFAP expression remained high in A-Rat AS after 24 h. CSPG is known to contribute to glial scar formation after injury, thus acting as a barrier against new axonal growth into the damaged site. However, in the *in vitro* scratch model, CSPG expression (shown in Fig 3E) was observed in all three groups, and did not show obvious variations after injury.

### Under normal culture conditions *in vitro*, gecko astrocytes had more cells in the S stage of the cell cycle and showed poorer migratory abilities than rat astrocytes

**Fig 4:**
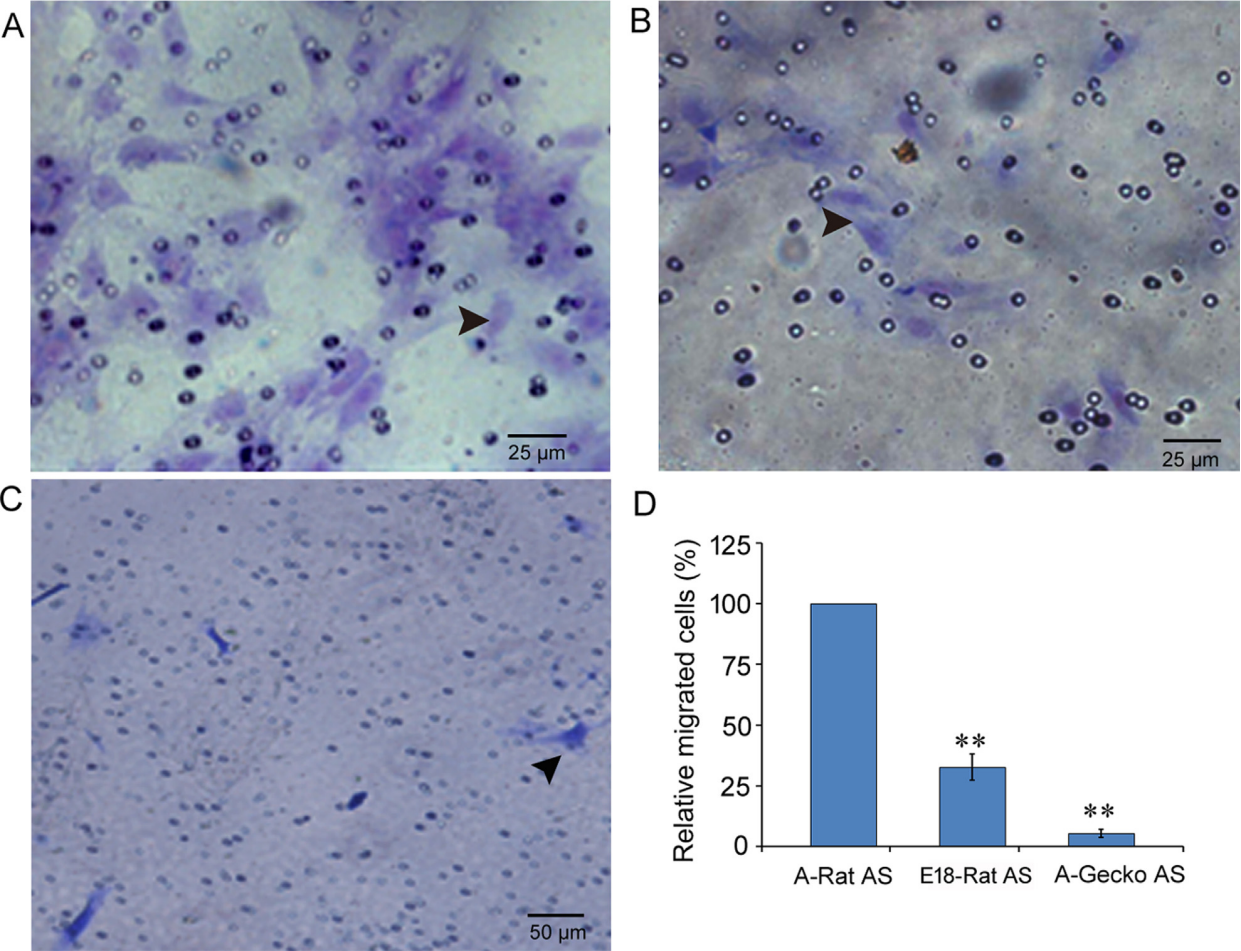
Under normal conditions, astrocytes from geckos showed poor migration abilities compared to astrocytes from rats. A–C show representative transwell images of cresyl violet staining in astrocytes from the adult rat (A-Rat AS) (A), embryonic rat E18-Rat AS (B), and adult gecko (A-Gecko AS) (C). (D) Graph showing transferred cells of three kinds of astrocytes, data are represented as the mean ± SD, compared to A-Rat AS, **p<0.01.

After the scratch injury, we observed that A-Rat AS proliferated and migrated more rapidly than A-Gecko AS. Thus, we wondered whether this was also the case under normal conditions. In order to address this, we performed flow cytometry and transwell assays to evaluate the cell cycles of astrocytes and their migration capacities under normal conditions. Cell cycle analysis showed that cultured astrocytes from adult rats, E18 rats, and adult geckos presented with 9.35% ± 0.03, 24.05%± 0.05, and 14.47% ± 0.07, respectively, of their cells in the S stage. In the transwell assay, the number of migrated astrocytes from adult rat spinal cords was about 3 times that of E18 rats and 18 times that of adult geckos (E18-Rat AS = 32.7% ± 5.4 and A-Gecko AS = 5.5% ± 1.8; shown in Fig 4). These results suggest that, under normal conditions, astrocytes from adult gecko spinal cords have higher proliferation and lower migration than astrocytes in rats. These results raised an interesting question. Given that A-Rat AS have lower proliferation abilities, but exhibit greater improvement in response to external stimuli, we wondered whether astrocytes from different species or different development stages possess different gene expression patterns.

### Transcriptome analysis of astrocytes from adult rats, adult geckos, and embryonic rats

RNA sequencing-based global transcriptome analysis provides a comprehensive view of the expression pattern of cells and tissues [24]. Here, we investigated the transcriptomes of astrocytes derived from adult rats, adult geckos, and E18 rat spinal cords, and aimed to determine differences in their expression profiles. We presumed that if differences were identified, they might contribute to the differential response to injury observed between the species.

**Fig 5:**
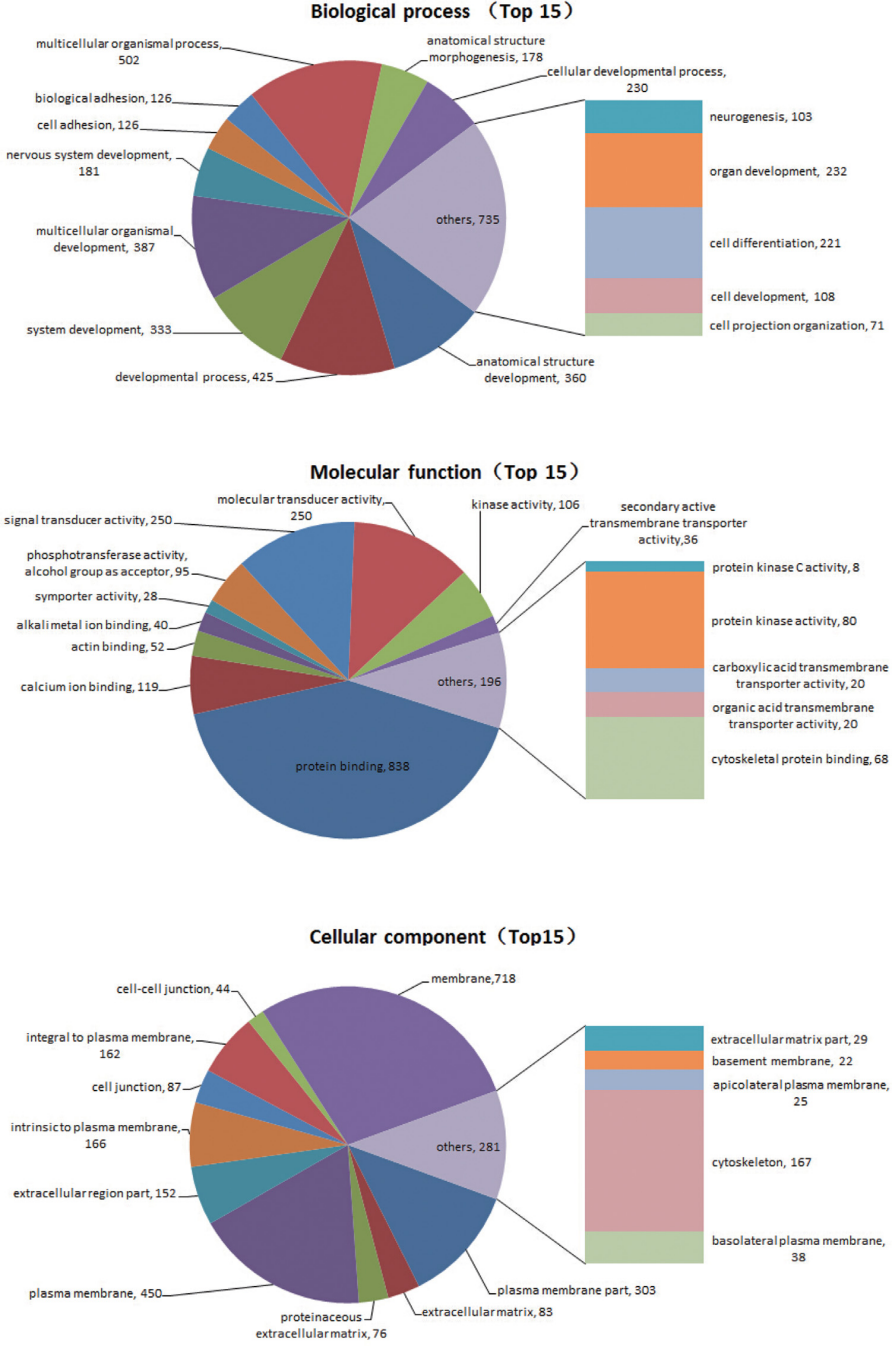
The gene ontology (GO) term enrichment analysis of differentially expressed genes (DEGs). DEFs between astrocytes from adult geckos, embryonic rats, and adult rats were examined. 5230 DEGs between the astrocytes from adult and embryonic rats were identified. Among these DEGs, 1585 genes showed higher or lower expression (more than 2-fold differences) in astrocytes from the adult rat versus astrocytes from the ebryonic rat and adult gecko. Pie charts show the GO term and its relation to various biological processes, molecular functions, and cellular components.

Comparing data across species is always challenging owing to differences in their genetic background. Therefore, comparisons were focused on DEGs between astrocytes from adult and embryonic rats. We also aimed to determine which of the DEG expression profiles (adult versus embryonic) was more similar to A-Gecko AS. We first identified 5230 DEGs between the astrocytes from adult and embryonic rats. Among these DEGs, 1585 genes showed higher or lower expression (more than 2-fold differences) in A-Rat AS versus E18-Rat AS and A-Gecko AS (S1 File). A KEGG analysis of these 1585 genes showed that the most frequently involved pathways were those relating to cancer, arrhythmogenic right ventricular cardiomyopathy, axon guidance, and tight junction formation (Table 1). Moreover, a GO term enrichment comparison revealed that the biological processes of development, cell adhesion, and responses to stimuli were significantly enriched in the DEGs (Fig 5). These data indicate that astrocytes from adult rats and *G*. *japonicus* respond differently because of intrinsic discrepancies. Moreover, these findings suggest that A-Gecko AS share characteristics with E18-Rat AS.

**Table 1:** The Kyoto encyclopedia of genes and genome-based (KEGG) pathway enrichment assay of differentially expressed genes between astrocytes from adult geckos, embryonic rats, and adult rats.

KEGG pathway		Gene counts
**Metabolism**		20
	Propanoate metabolism	8
	Inositol phosphate metabolism	12
**Environmental Information Processing**		117
	Wnt signaling pathway	28
	Hedgehog signaling pathway	11
	TGF-beta signaling pathway	17
	Phosphatidylinositol signaling system	17
	ECM-receptor interaction	19
	Cell adhesion molecules (CAMs)	25
**Cellular Processes**		94
	***Focal adhesion***	***36***
	Adherens junction	17
	Tight junction	26
	Gap junction	15
**Organismal Systems**		97
	***Leukocyte transendothelial migration***	***28***
	Vascular smooth muscle contraction	21
	Aldosterone-regulated sodium reabsorption	9
	Long-term depression	13
	***Axon guidance***	***26***
**Human Diseases**		184
	***Pathways in cancer***	***57***
	Colorectal cancer	15
	Glioma	12
	Melanoma	14
	Small cell lung cancer	15
	Non-small cell lung cancer	12
	Hypertrophic cardiomyopathy (HCM)	19
	***Arrhythmogenic right ventricular cardiomyopathy (ARVC)***	***21***
	Dilated cardiomyopathy (DCM)	19

Differentially expressed genes (DEGs) between astrocytes from adult and embryonic rats were identified first. Among these DEGs, 1585 genes were further investigated for higher or lower expression levels (more than 2 folds) in astrocytes from adult and embryonic rats, as well as astrocytes from adult geckos. The table shows the most frequently involved pathway of these 1585 genes (determined by the KEGG analysis). Italicized bold pathways belong to the top five most frequent involved pathways.

**Fig 6:**
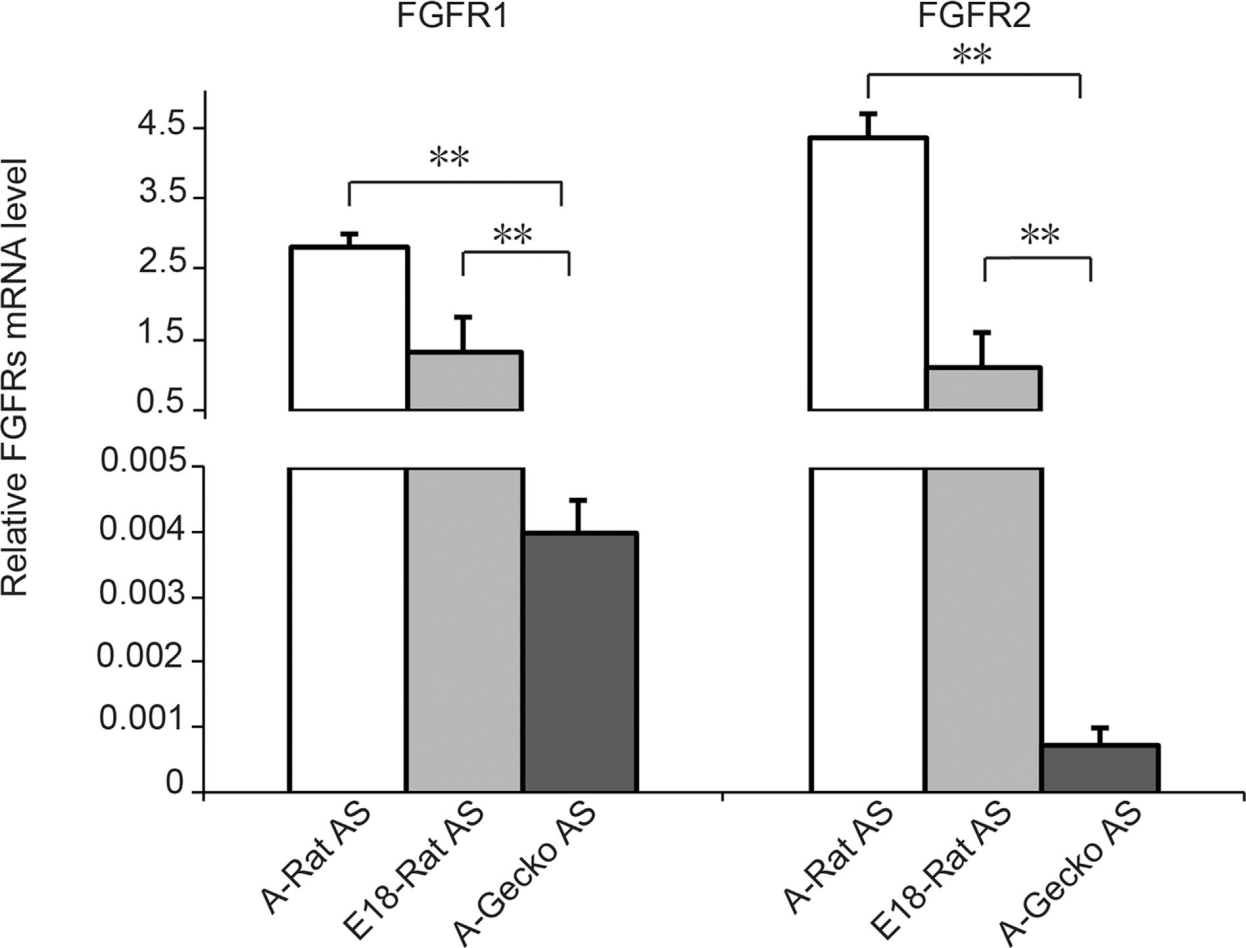
Relative fibroblast growth factor receptor 1 (FGFR1) and FGFR2 mRNA expression. The expression of FGFR1 and FGFR2 in cultured adult rat astrocytes (A-Rat AS), embryonic rat astrocytes (E18-Rat AS), and adult gecko astrocytes (A-Gecko AS) was examined. The *EF1α* gene served as an internal control. Graphic representation of the real-time PCR results in different astrocytes; **p < 0.01.

Cell signaling via fibroblast growth factor receptors (FGFRs) is known to mediate a variety of cellular responses including cell proliferation, differentiation, and migration [25]. Additionally, to confirm the RNA sequencing-based global transcriptome analysis, we chose FGFR1 and FGFR2 as the representative genes involved in scar formation/proliferation, and we confirmed their changes using real-time quantitative PCR (Fig 6). All primers were list in Table 2. The results showed that the expression of FGFR1 or FGFR2 mRNA in A-Gecko AS is lower than the expression of FGFR1 and FGFR2 in A-Rat and E18-Rat AS, which is consistent with the data in the RNA sequencing-based global transcriptome analysis. The gene expressions of three kinds of astrocytes were evaluated by the value of the reads per kilobase of transcript per million reads (RPKM). The RPKMs of FGFR1 were 154.2603955, 52.41731918, and 12.38431737 in A-Rat AS, E18-Rat AS and A-Gecko AS respectively, and those of FGFR2 were 26.09732716, 6.926015988, and 0.594580287 in the above astrocytes (supplied in S1 File).

**Table 2:** DNA sequences of primers used for real time quantitative PCR in this study.

Gene Name	Sequence (5'→3')
Rat *EF1α* sense	5'- gcggggacaagaaggtcat -3’
Rat *EF1α* antisense	5'- tacggcagtctggtgtacaaat-3’
Rat *FGFR1* sense	5'- gcacctgaggcattgtttga -3'
Rat *FGFR1* antisense	5'- gcttgtccattcgatgaccc -3'
Rat *FGFR2* sense	5'- gcagttggtggaagacttgg -3'
Rat *FGFR2* antisense	5'- ataaggcatggggtctggag-3'
Gecko *EF1α* sense	5'- gatggaaagtgacccgca -3'
Gecko *EF1α* antisense	5'-gaggaagacgcagaggtttg-3'
Gecko *FGFR1* sense	5'-ggtcctttggtgttctgctg -3'
Gecko *FGFR1* antisense	5'-gaaggaacagcatgccaaca -3'
Gecko *FGFR2* sense	5'-gcagttggtggaagacttgg -3'
Gecko *FGFR2* antisense	5'-ataaggcatggggtctggag -3'

## Discussion

Lower vertebrates and developing higher mammals have the potential to regenerate injured or lost tissues, including tissues within the central nervous system. However, this capability decreases with evolution and development, and is thus very limited in adult mammals. Moreover, the underlying mechanisms of tissue regeneration are still poorly understood. Previous studies have indicated that the presence of more progenitor cells, less cell death, and fewer glial scars increase the regenerative capacity after spinal cord injury in adult lower- and embryonic higher-vertebrates [9, 26]. Glial scarsare composed of activated astrocytes and inhibitory matrix molecules. In the current study, we compared the activation and proliferation of astrocytes from adult rats, embryonic rats, and adult geckos after *in vitro* injury. We found that A-Rat AS were much more sensitive to injury than E18-Rat AS and A-Gecko AS. While the molecular mechanisms of astrogenesis are not completely understood, it is widely accepted that external factors such as cytokines (small molecules released after cell injury) are the major cause of glial scar formation [27]. Moreover, it has been shown that these molecules can directly or indirectly lead to astrocytic activation. Our study indicated that the properties of astrocytes are important for astrogliosis, as astrocytes can initiate astrogliosis. To our knowledge, this is the first comparative study of astrogliosis between mammals and reptiles. Moreover, we found that the phylogeny and developmental stage both showed discrepant astrocytic responses to an external stimulus. These results suggest that modulating the astrocytic response could prove useful for treating injuries to the central nervous system.

To further illustrate the intrinsic properties of astrocytes from the various animal models, we performed transcriptome analyses and found that A-Gecko AS shared similar expression patterns with E18-Rat AS. GO term and KEGG pathway enrichment analyses of astrocytic DEGs revealed that these genes were involved in cellular behaviors such as migration, proliferation, and the response to stimuli. These data support the view that astrocytes from lower species exhibit different responses to injury. Moreover, this discrepancy could explain the greater regenerative capacity of some animals after spinal cord injury. The various properties of the astrocytic response to injury revealed in this study are extremely compelling, especially when compared to astrocytes under normal conditions. Thus, future studies will need to validate the roles of the DEGs obtained from activated astrocytes in order to employ these genes to *in vitro* and/or *in vivo* models of astrogliosis.

## Supporting Information

S1 File DEGs of 1585 genes, KEGG analysis and GO term enrichment in A-Rat AS versus E18-Rat AS and A-Gecko AS. (XLSX)
